# Management of High-Risk Atherosclerotic Patients by Statins May Be Supported by Logistic Model of Intima-Media Thickening

**DOI:** 10.3390/jcm10132876

**Published:** 2021-06-29

**Authors:** Dorota Formanowicz, Jacek B. Krawczyk, Bartłomiej Perek, Dawid Lipski, Andrzej Tykarski

**Affiliations:** 1 Department of Medical Chemistry and Laboratory Medicine, Poznan University of Medical Sciences, 60-806 Poznan, Poland; 2 School of Mathematics & Statistics, The University of Sydney, Sydney, NSW 2006, Australia; jacek.krawczyk@sydney.edu.au; 3 Department of Cardiac Surgery and Transplantology, Poznan University of Medical Sciences, 61-001 Poznan, Poland; bperek@ump.edu.pl; 4 Department of Hypertension, Angiology and Internal Disease, Poznan University of Medical Sciences, 61-001 Poznan, Poland; dlipski@ump.edu.pl (D.L.); tykarski@o2.pl (A.T.)

**Keywords:** atherosclerosis, statins, control-theoretic model, logistic growth

## Abstract

While the use of statins in treating patients with atherosclerosis is an undisputed success, the questions regarding an optimal starting time for treatment and its strength remain open. We proposed in our earlier paper published in Int. J. Mol. Sci. (2019, 20) that the growth of intima-media thickness of the carotid artery follows an S-shape (i.e., logistic) curve. In our subsequent paper in PLoS ONE (2020, 15), we incorporated this feature into a logistic control-theoretic model of atherosclerosis progression and showed that some combinations of patient age and intima-media thickness are better suited than others to start treatment. In this study, we perform a new and comprehensive calibration of our logistic model using a recent clinical database. This allows us to propose a procedure for inferring an optimal age to start statin treatment for a particular group of patients. We argue that a decrease in the slope of the IMT logistic growth curve, induced by statin treatment, is most efficient where the curve is at its steepest, whereby the efficiency means lowering the future IMT levels. Using the procedure on an aggregate group of severely sick men, 38 years of age is observed to correlate with the steepest point of the logistic curve, and, thus, it is the preferred time to start statin treatment. We believe that detecting the logistic curve’s steepest fragment and commencing statin administration on that fragment are courses of action that agree with clinician intuition and may support decision-making processes.

## 1. Introduction

Knowledge of the intima-media (IMT) growth process is essential for decision making regarding statin therapy initiation and intensification. The purpose of our study is to assess whether the mathematical modeling of IMT growth, proposed in [1,2], can assist clinicians at crucial stages of the process.

Thickening of the intima-media complex, which is an undisputed symptom of atherosclerosis, is an inevitable consequence of the process of aging of the human vascular system. The age-related changes can be observed at both micro- and macro-levels. At the micro-level, cellular senescence manifests as reduced cell proliferation, an irreversible arrest of growth, apoptosis, DNA damage, etc. [3]. At the macro-level, atherosclerotic plaques with calcium deposits can be detected by imaging examinations applied routinely in clinical practice; see [4]. The deposits are of clinical significance, as they are the final stage of vascular degeneration.

Clearly, many factors contribute to atherosclerosis development; see e.g., [5]. In particular, there are some inherited predisposing factors and other factors that may be modified by our lifestyle, which include diet; physical activity; and adherence to recommendations of optimal management of many atherosclerosis-modifying diseases, such as arterial hypertension, diabetes, and hyperlipidemia—see [6].

According to our knowledge, it is very difficult, if at all possible, to regress atherosclerotic plaque development. However, in some cases, doctors are able to stabilize the plaque and, therefore, inhibit disease progression. This can happen if intensive treatment with high-dose statins is applied; see [7].

However, doubts surrounding the dosage of statins and the therapy timing remain. A few years ago, a cohort study [8] on the general UK population reported statin overuse in patients with low cardiovascular risk and underuse in patients with high cardiovascular risk. Furthermore, another study (see [9]) points to possible adverse events, such as muscle and liver injury, cognitive impairment, new-onset diabetes mellitus, and even hemorrhagic stroke, as a result of long-term statin therapy. Moreover, there is no strong clinical evidence that the elderly would benefit from statin therapy; see [10]. Statin therapy should be individualized and based on the patient’s risk profile. This study uses a model that may alleviate the above concerns.

The measurement of the carotid artery IMT can be achieved by the simple and noninvasive technique of measuring atherosclerotic burden [11]. Consequently, IMT has been utilized as a reliable marker of drug efficiency and tested in clinical trials devoted to atherosclerosis treatment. Moreover, IMT is widely accepted as a screening tool that can be used together with the traditional risk factors assessment. The size and dynamics of IMT can help in determining optimal therapeutic interventions as well as in the application of other diagnostic tools; see [12]. Although there are studies that have presented a discrepancy between carotid IMT changes, prognosis, and the course of cardiovascular pathologies [13,14], the overwhelming clinical data (see [15,16,17,18,19]) strongly confirm that the IMT will continue to be used as a valuable tool in clinical research.

It was conjectured in [1] that the IMT growth process follows an S-shape (i.e., *logistic*) curve. An application of a mathematical model based on this proposition to atherosclerosis management by statins was developed in [2]. However, the number of observations in [1] of the logistic model being calibrated was low (27); therefore, the quantitative reasoning based on that model was mainly of conceptual value rather than being immediately applicable to management of atherosclerosis. The recent availability of the large Cardio Poznan Database (122 observations) [20] has created an opportunity for us to perform a new calibration of the logistic model from [1] and provide some managerial advice.

In the next section, we describe briefly the Cardio Poznan Database, the source of our new data. Then, Section 3 discusses the new data support for the S-shaped IMT growth. Subsequently, in Section 4, we propose a procedure for inferring an optimal age to start statin treatment for a particular group of patients. The paper ends with brief Concluding Remarks and an Appendix, which contains a few summary statistics concerning the observations gathered in the database.

## 2. Cardio Poznan Data

The data collection project, see [20], received a positive opinion (decision No. KB 341/21) of the Bioethics Committee of the Poznan University of Medical Sciences.

The data collection involved 122 consecutive patients: 78 males (63.9%) and 44 females (36.1%). Their mean age was 49.6 years, and the standard deviation was 15.6 years. These patients were treated in the Department of Hypertension and Angiology and Internal Medicine at the Poznan University of Medical Sciences in the first quarter of 2020. From this group, we selected the male subjects (*n* = 31) who had arterial hypertension-related cardiac disease, i.e., coronary artery disease (CAD) and/or vascular complications, such as peripheral vascular disease (PVD). This group of 31 *male* patients represents the observation sample for our study. These patients are referred to as *severely sick men*. They are split into two subgroups: (1) patients undergoing statin therapy, denoted as *statin(+)*, and (2) patients not treated with statins, denoted as *statin(-)*.

We provide a summary of the demographic and clinical data of the studied patients in Appendix A, Table A1. The findings of the laboratory tests and imaging examinations (carotid artery Doppler ultrasonography) are provided in Table A2.

## 3. Support for S-Shaped Growth of the Atherosclerotic Plaque

### 3.1. Importance of S-Shaped Growth for Atherosclerosis Treatment

Confirming both, the S-shape of the IMT growth process (see [1,2]) and its quantitative features is important for clinical reasons. Notably, knowledge of the S-shape model for the atherosclerosis process will enable us to indicate the patient age ranges of the disease’s fast and slow growth. Specifically, we aim to identify when the atherosclerotic process is fast. In an attempt to prevent the IMT from thickening, the ideal place (here, the age range) to start statin treatment is when the curve is steep, i.e., before it eventually flattens at the patient’s older age. Below, we present why we think this line of thought could help clinicians.

A slow IMT growth can occur when the patient is very young—too early to administer statins—or when the patient is very old (see [21])—too late to start treatment. Assuming statin treatment slows down the plaque growth by a certain amount, if this amount is subtracted from fast growth, the disease-slowing effect will be more substantial than if statins are administered when the disease progresses slowly. A mathematical explanation for this effect involves the model of nonlinearity. This emulates nonlinearity of the underlying medical process, as postulated in [1]. With the help of new data, we aim to confirm the logistic growth of IMT and establish the steepest fragment on an IMT growth model.

### 3.2. The S-Shape Conjecture

The S-shape of the atherosclerosis process was postulated in [1] (see also [2]). Regrettably, the number of data points available in [1] is low, and the proposed model’s goodness-of-fit statistics are mixed. In an attempt to improve the statistical significance of the model, we now use a larger dataset [20] and carry out a new parameter identification procedure for the logistic model of atherosclerosis.

**Figure 1:**
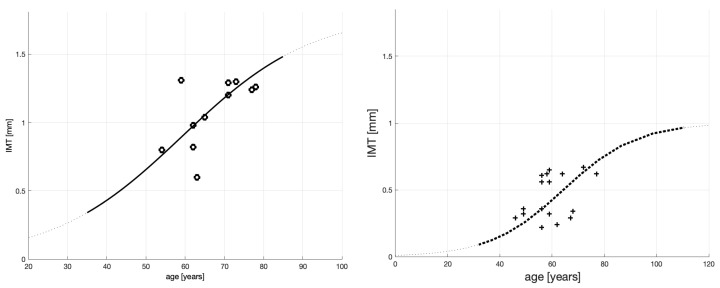
Fitting of the IMT time profile for severely sick patients (on dialysis)—**left panel** (11 observations), and fitting of the IMT time profile for healthy patients—**right panel** (16 observations).

The starting point of our analysis of atherosclerosis in [1], continued in [2], is Figure 1 presented below (produced out of Figures A1 and A2 published in [1]). The figure panels show S-shaped curves calibrated ibidem using our previous data on IMT vs. age of severely sick men on dialysis.

The S-shaped curves in Figure 1 are of the following analytic form:

(1) $$x\left(t\right)=\frac{cx_{0}e^{at}}{c+x_{0}\left(e^{at}-1\right)}\;.$$

Each is a solution to the logistic differential equation $\frac{dx}{dt}=ax\left(1-\frac{x}{c}\right),x\left(0\right)=x_{0}$ where

- $x_{0}$ is an initial condition;
- *c* (called “carrying capacity” in population dynamics) is the terminal size of IMT toward which a patient’s plaque size converges, given this patient’s overall health level; presumably, a severely sick patient will have a large *c* and if, eventually, IMT = *c*, the artery will not be able to handle this plaque thickness and the patient will pass away;
- *a* determines the speed of plaque buildup.

The difference in the appearances of the S-shaped curves is attributed to the patients’ medical conditions. Typically, patients whose medical conditions require dialysis will have a large *c* that will grow more steeply. This is why the left panel curve grows faster and reaches higher values. The model coefficients for the severely sick men on dialysis (see the left panel) are provided in Table 1. (The model coefficients for the healthy patients’ behavior are omitted, because healthy patients are not considered in this paper.)

The evidence provided by the curves, together with the existing clinical literature cited in [1], led us to propose ibidem that the atherosclerotic plaque’s growth over a patient’s life span has an S-shape and can be represented mathematically by a logistic function.

As mentioned above, the number of data points in Figure 1 is low, and the curves’ goodness-of-fit statistics, reported in [1] and cited in Table 1, are mixed. For example, while the coefficient of determination $R^{2}=0.36$ for the case of severely sick patients might be considered satisfactory, the model-corresponding values of SSE = 0.389 and RMSE = 0.1881 are ordinary for such a small data sample of 11 observation points. Nevertheless, we conjectured ibidem that in aggregate, the above goodness-of-fit statistics provide support for a logistic process of IMT formation. However, given the low number of observations, they do not carry sufficient weight for the obtained model to be relied upon in clinical diagnostics and treatment, which are our ultimate goals of the model usage (see [2]).

## 4. New Data Support

### 4.1. Patient Aggregate

**Figure 2:**
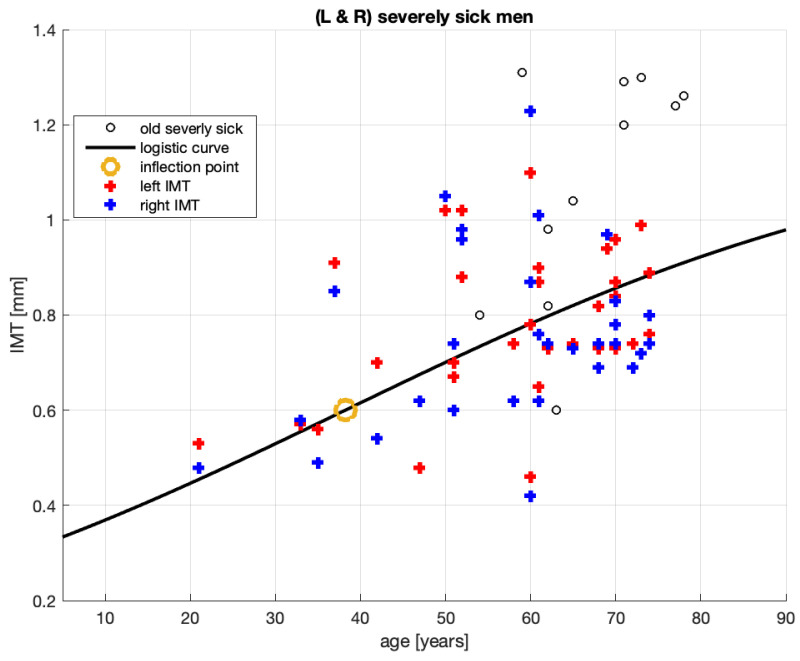
Fitting of the IMT time profile for severely sick men from [20].

Figure 2 shows the new 62 IMT measurement points from [20] for the left and right arteries of severely sick men (see the legend for points marked L and R). The 11 black circles are the same as those in the left panel of Figure 1 and represent the 2008–2011 sample ([22]) of the severely sick men on dialysis. Their IMT values point to accelerated atherosclerosis.

The data from [1] did not distinguish between the left and right arteries; thus, it is likely that both were represented. Furthermore, both datasets are *aggregates* of sick patients who take statins (for different period lengths) and sick patients who do not take statins. Hence, from the point of view of gravity of the sickness, the old and new data may be compatible.

We used the new data to obtain the logistic curve in Figure 2. The identified parameters of this curve are provided in Table 2.

There are several comments to make concerning Figure 2.

1. The 2008–2011 population of sick men on dialysis (shown in Figure 1 left panel; see also the black empty circles in Figure 2) must indeed have been composed of sicker patients than those in the current sample. The black empty circles with a value of IMT over IMT ≥ 1.2 (mm), for example, constitute more than 50% of the whole sample; among the blue and red crosses (current sample), only 1 in 62 exceeds this value.
2. The old data spread of patient ages (horizontal axis) is much smaller (53–78 years old) than that of the new data (19–74 years old). This, combined with the fairly large spread of the old-sample IMT values (vertical axis), means that the old curve’s steepness ( Figure 1) should be greater than that of the new one (Figure 2). Indeed, we computed the slope on this curve to be between 58 and 67 years old, and it is more than three times larger than the maximum slope reported in Table 2 (i.e., 0.0265 vs. 0.0086). This is consistent with the severity of *accelerated* atherosclerosis among the-old data patients.
3. We note that it should be easier to fit a 3-parameter (logistic) curve to 9 observations than to 62. Therefore, the distance between an observation point and its model should be shorter in the old model, but it is not. Remarkably, the RMSE of the new model is smaller than that of the old one. This indicates that the logistic model for the new data guarantees a smaller mean distance between an observation point and its logistic model’s value. Therefore, in terms of these distances, the new logistic model is better aligned with the new data than the old model was with the old data.
4. The new model $R^{2}$ is smaller than that of the old model, which is an undesirable result. However, small or even negative values of $R^{2}$ may occur when fitting non-linear functions to data. Our model is non-linear. Therefore, $R^{2}$ alone cannot be used to judge how good, or bad, our model is.

**Proposition** **1.** *We propose that, on balance, for small RMSE (good) versus small $R^{2}$ (bad), the logistic curve in Figure 2 supports the conjecture of the authors of [1] regarding the S-shaped process of the IMT growth.*

**Proposition** **2.** *The steepest part of the logistic curve is around its inflection point (≈38, 0.6; see Table 2 and the beige circle on the curve in Figure 2). Therefore, propose that for the group of sick men, starting patient medication at around 38 years old may be the most beneficial approach.*

The new dataset (from [20]), which we use in this study, concerns severely sick patients. However, this set is an aggregate of many patient types. The data are *inhomogeneous* in (at least) two aspects. First, they contain statin-medicated and statin-non-medicated patients. Second, the medicated patients take various doses of different statins (atorvastatin and rosuvastatin) for a varying number of months. Of course, model coefficients crucially depend on the sample patients’ conditions. For example, the 2008–2011 data [22] of the severely sick men *on dialysis* generated a steeper S curve than that of the new data. Arguably, models built for a homogeneous patient group should be more reliable than a model built for a patient aggregate. In the next sections, we *disaggregate* the severely sick patient group into non-medicated and medicated patient subgroups and propose an IMT growth model for each subgroup.

### 4.2. Non-Medicated, Severely Sick Men

The group analyzed here is composed of patients who are severely sick but remain non-medicated. We refer to this subset of patients classified in the database [20] as *non-medicated*.

We now analyze the IMT growth process of the non-medicated patients.

**Figure 3:**
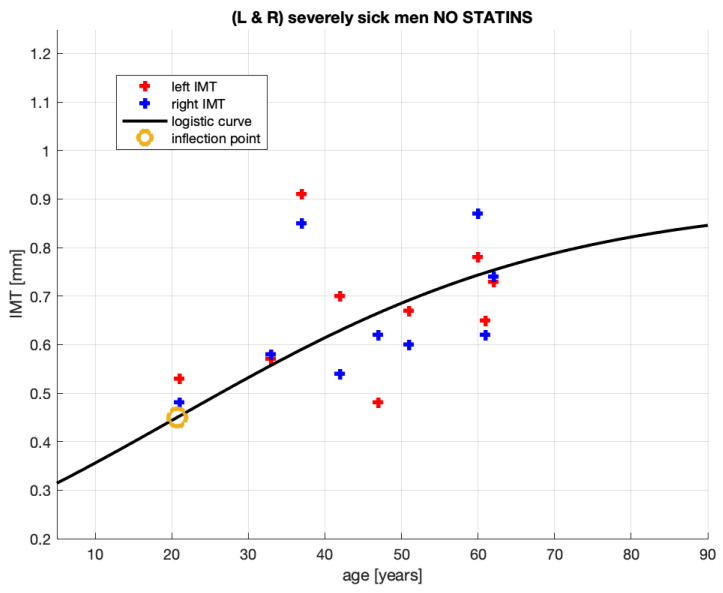
Fitting of the IMT time profile for non-medicated, severely sick men from [20].

Figure 3 shows 18 pairs (age and IMT) of the measurement points for severely sick, non-medicated men from [20]. Of course, these points are also represented in Figure 2. Now, they are analyzed alone.

The parameters of the logistic curve in Figure 3, which represents the IMT growth model, are provided in Table 3.

Observing Figure 3, it can be noted that, in comparison to that of Figure 2, the logistic curve stabilizes here at a much lower IMT value. This may suggest that these patients’ atherosclerotic plaque has been developing in a different manner to that of the other patient aggregate. This is discussed further in Section 4.1.

These patients are likely to have stable atherosclerotic plaque and be free from many of the clinical symptoms recorded in [20]. In particular, they may have developed collateral circulation, hence remaining in a clinically stable condition. Moreover, the IMT measurements, consequential for our study, are the IMT’s thickness quantifications only. However, using the thickness measurement only, it is impossible to conclude the morphology of the atherosclerotic plaque. Nevertheless, it may be the morphology that is relevant to the classification of a patient as *severely sick*. For better insight into these statin-non-medicated patients, their characteristics, demographic data, and clinical and laboratory data are compared with those of severely sick but statin-medicated patients in Table A2.

While this group of non-statin-medicated patients might not profit from statin treatment, the observations of their IMT growth, as shown in Figure 3 and Table 3, assist in furthering the discussion of the growth’s S-shape.

5. As per the goodness-of-fit statistics, the obtained model for the non-medicated, severely sick men is more reliable than that for the aggregate of the severely sick men. In particular, RMSE shrunk from 0.1664 mm for the aggregated patient group to 0.1263 mm for the non-medicated group. This means that the root mean square error (RMSE)—practically, the expected distance between an actual measurement and the corresponding model value— diminished by about 25%.
6. SSE and $R^{2}$ also improved (the latter only marginally).
7. The observations of [5,6] were expected. As previously mentioned, the non-medicated, severely sick men constitute a more homogeneous group than the severely sick aggregate of which they are a subset. Arguably, more uniformity in patient conditions will improve the goodness-of-fit statistics.

We draw the information on qualitative and quantitative properties of the IMT growth process in the non-medicated patients from Figure 3 and Table 3 as follows:

8. Patient age when the plaque growth is maximum (see the inflection points marked by the beige circles in each figure) differs between the groups. The maxima are 38 and 21 years old
9. The non-medicated patient model’s $R^{2}$ is slightly larger than that of the aggregate group. We cannot though dwell on this improvement, however, since both models’ determination coefficients are very small.

**Proposition** **3.** *The above comments lead to the proposal that the model for the non-medicated, severely sick men (see Figure 3 and Table 3) supports the conjecture of the authors of [1] regarding the S-shaped process of the IMT growth.*

### 4.3. Statin-Medicated, Severely Sick Men

The complement to the non-medicated, severely sick men within the severely sick men aggregate in [20] is the group of severely sick men receiving statins or *statin-medicated* men. We now analyze the IMT growth process of these statin-medicated patients.

**Figure 4:**
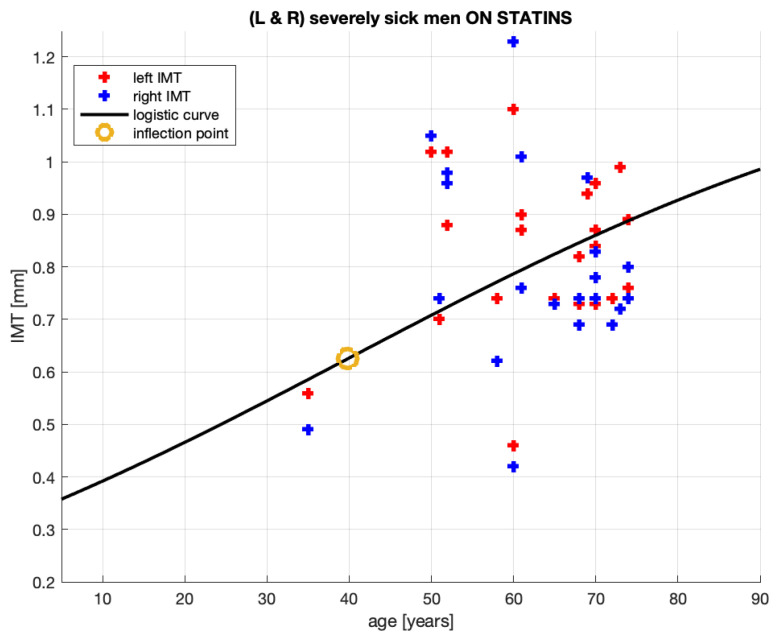
Fitting of the IMT time profile for statin-medicated, severely sick men from [20].

Figure 4 shows 44 pairs (age, IMT) of the measurement points for severely sick statin-medicated men from [20]. Of course, these points are also represented in Figure 2. Now, they are analyzed alone.

The parameters of the logistic curve in Figure 4, which represents the IMT growth model, are provided in Table 4.

Below are our comments regarding the model for the statin-medicated, severely sick men.

**Figure 5:**
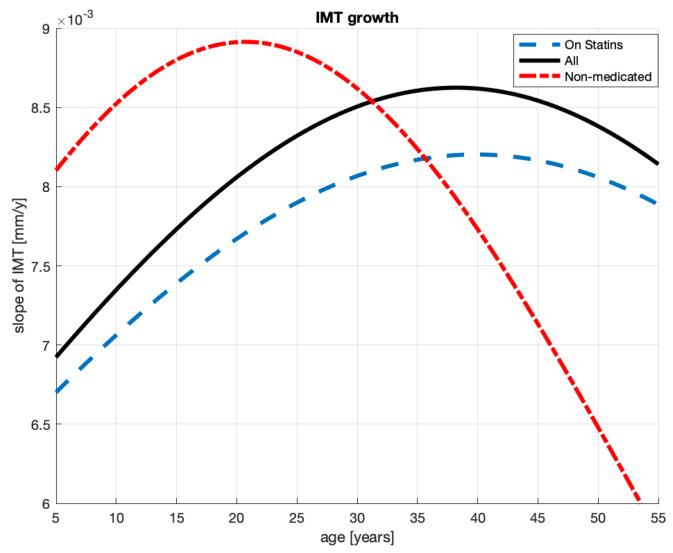
The slopes of the plaque formation processes for the severely sick men on statins, without statins, and their aggregate.

10. The group analyzed here, while extracted from the severely sick men aggregate, is not necessarily composed of patients of much the same conditions. It is composed of patients who are severely sick and receive statins. Some patients in this group receive atorvastatin, some others—rosuvastatin, two not identical statin medications; the treatment periods vary between 2 and 36 months; the doses vary between 5 and 40 [mg]. This implies that—in this group—some patients may have suffered from an advanced stage of atherosclerosis mitigated by statins administered for short or for long periods. Undoubtedly, these inhomogeneities will complicate obtaining a reliable *quantitative* relationship between the age and plaque thickness formation.
11. Observing Figure 4, it can be noted that, generally, the logistic curve appears to be very similar to the curve obtained for the patient aggregate in Figure 2. This includes the suggested ages for starting statin treatment, which are 40 and 38.
12. The similarities are not unexpected given that, from the 64-patient measurements in Figure 2, only 18 were subtracted as non-medicated (see Figure 3). The parameters of the logistic curves are also similar.
13. Qualitatively, the model corresponds quite well to the intuition we may have about this patient group. To be medicated, their initial conditions $x_{0}$ should be worse than in the aggregate of the medicated and non-medicated patients. Indeed, the $x_{0}$ levels are 0.325>0.3, where the first number is for the statin-medicated group and the second is for the aggregate. Their *c* levels 1.25>1.2 suggest that should these patients have remained non-medicated, the plaque would have grown larger in the medicated group than in the aggregate. The patients *are* being medicated and, as a result of that, the plaque grows at a slower pace for these patients than for the patient aggregate. See the coefficients *a* and the maximum slopes document.
14. The slower plaque growth in the medicated patients is also observed in Figure 5: the dash-dotted (blue) line remains below the solid (black) line, where the latter corresponds to the patient aggregate.
15. The goodness-of-fit statistics of the statin-medicated patient model do not suggest that this model is better than the one proposed for the patient aggregate. Although SSE has improved (1.369<1.689), the expected distances between an actual measurement and the corresponding model’s value (RMSE 0.1784>0.1664) and $R^{2}$ have worsened. We remind the reader that even $R^{2}<0$ should not alone disqualify a nonlinear model.

**Proposition** **4.** *The logistic growth model (see Figure 4 and Table 4) for the statin-medicated, severely sick men has an explanatory value in that it helps to explain the IMT plaque formation process for this group of patients. However, the goodness-of-fit statistics for this model do not indicate that this model is an improvement on the patient aggregate’s model referred to in Proposition 1.*

## 5. How to Infer an Optimal Age for Starting Statin Treatment

We have previously proposed that the optimal patient age for a specific group of patients to start statin treatment is when the curve is at its steepest. This seems the best locus on the S-curve to prevent it from rising or, in clinical terms, to prevent IMT from thickening. Figure 1, Figure 2, Figure 3 and Figure 4 can help to find such loci for a specific group of patients.

The patterns of the speed of plaque formation differ between the aggregate of the severely sick patients and the non-medicated patients. We can see these speeds in Figure 5: the solid line represents the patient aggregate and the dash-dotted line the non-medicated patients. The dashed line indicates patients on statin; see the next section.

The maximum speed of the plaque formation for the non-medicated patients (a) is higher than the patients’ aggregate i.e., 0.008915>0.008625 for the former and latter group and (b) occurs 17 years earlier for the former and latter groups.

We claimed in Proposition 2 that 38 years of age (see infl. point in Table 2) is the right age to start statin treatment in the aggregated group. Our argument is that a decrease—induced by treatment—in the slope of the IMT logistic curve is most efficient when the curve is at its steepest, whereby the efficiency concerns lowering the future IMT levels. Beginning treatment of non-medicated patients at the young age of 21 years old (see infl. point in Table 3) appears to be early. However, as judged by the logistic curve in Figure 3, these patients do not need medication. Arguably, their own bodies manage to considerably slow down plaque growth at this age. Just after the inflection point, we can see in Figure 5 that the dash-dotted (red) line quickly drops far below the other lines. The corresponding graph of the plaque formation in Figure 3 flattens as if these non-medicated patients were submitted to treatment. In fact, one could claim that it is their own bodies that generate this plaque formation pattern.

Briefly, an analysis of IMT slopes can help in making decisions regarding the best patient age for commencing statin treatment. The steepest slope can be learned from the slope’s first derivative graph (see Figure 5). The steepest slope is where the derivative attains a maximum.

## 6. Limitations and Strength of This Study

An important limitation of our study is that it concerns male patients only. The reason for this is that the female population was less represented in [20]. Furthermore, the female patient population is less homogeneous than the male patient population in that their symptoms associated with cardiovascular pathologies are more variable and therefore more difficult to calibrate in the model than those of male patients.

Another limitation is related to the patient sample size in [20]. Although significantly larger than that in [1], our sample size is modest when compared with that of international studies; see e.g., the JUPITER trial [23]. With more patients in the database, perhaps augmented by a population-based study, we would be able to attempt to model IMT growth in female patients.

Notwithstanding these limitations, we strongly believe that the male patient sample size we used in this study was sufficient to validate the model proposed and explained in [1,2]. The results of the biostatistical data analyzed in this paper should assure clinicians regarding our model’s usefulness.

## 7. Concluding Remarks

Our study showed that logistic models of IMT growth can support clinician decisions concerning the use of statins in the treatment of atherosclerosis. Specifically, we suggest that the steepest segment of the IMT-growth’s S-shape curve, obtained for a specific group of patients, can be recommended as the appropriate disease phase to commence treatment. In numerical terms, we identified some statistical evidence that 38 years old may be an appropriate age to start treatment for the group of severely sick men in [20].

## Figures and Tables

**Table 1:** Parameters of the IMT time profile model for severely sick patients (on dialysis); see [1]. SSE is the sum of squared estimate of errors, and RMSE is the root mean square error. $R^{2}$ is the coefficient of determination.

$x_{0}$	*c*	*a*	SSE	RMSE	$R^{2}$	Sample Size
0.05	1.8	0.06	0.389	0.1881	0.36	11

**Table 2:** Parameters of the IMT growth model for severely sick men; see [20] (infl. point stands for the inflation point, and max slope indicates the fastest growth of IMT in mm/y for this group of patients).

$x_{0}$	*c*	*a*	SSE	RMSE	$R^{2}$	Infl. Point	Max Slope	Sample Size
0.3	1.2	0.02875	1.689	0.1664	0.0526	[≈38, 0.6]	0.008625	62

**Table 3:** Parameters of the IMT growth model for non-medicated, severely sick men from [20].

$x_{0}$	*c*	*a*	SSE	RMSE	$R^{2}$	Infl. Point	Max Slope	Sample Size
0.275	0.9	0.03962	0.2713	0.1263	0.0563	[≈21, 0.45]	0.008915	18

**Table 4:** Parameters of the IMT growth model for statin-medicated, severely sick men; see [20].

$x_{0}$	*c*	*a*	SSE	RMSE	$R^{2}$	Infl. Point	Max Slope	Sample Size
0.325	1.25	0.02625	1.369	0.1784	−0.13	[≈40, 0.625]	0.008203	44

## Data Availability

Data are available at https://www.researchgate.net/publication/351355752_CARDIO_POZNAN_DATA (accessed on 25 June 2021).

## Appendix A. CARDIO POZNAN DATA—Summary Statistics

We refer the reader to the Excel file at [20] for our field observations. Here, we present a few summary statistics concerning the observations.

**Table A1:** Summary of demographic and clinical CARDO POZNAN DATA severely sick male patients.

	All [*n* = 31]	Statin(−) [*n* = 9]	Statin(+) [*n* = 22]
Age [years]	58.3 (13.4)	47.4 (14.9)	62.6 (10.2)
70 years +	8 (25.8)	0	8 (36.4)
Height [m]	1.77 (0.08)	1.83 (0.08)	1.75 (0.07)
Weight [kg]	92.2 (16.7)	106.4 (21.3)	96.2 (13.9)
BMI [kg/m^2^]	32.3 (4.8)	32.7 (6.5)	32.2 (4.1)
Obesity (BMI > 30 kg/m^2^)	19 (61.3)	5( 55.6)	14 (63.6)
CAD	18 (58.1)	1 (11.1)	17 (77.3)
ACS in history	7 (22.6)	0	7 (31.8)
PVD	16 (51.6)	2 (22.2)	14 (63.6)
Cerebral *	16 (51.6)	2 (22.2)	14 (63.6)
stroke	2 (6.5)	0	2 (9.1)
Lower extremities	5 (16.1)	0	5 (22.7)
CKD **	7 (22.6)	3 (33.3)	4 (18.2)
COPD	3 (9.7)	1 (11.1)	2 (9.1)
Thyroid diseases	2 (6.5)	1 (11.1)	1 (4.5)
GI disorders	1 (3.2)	1( 11.1)	0
Active smokers	9 (29.0)	3 (33.3)	6 (27.3)
DM	12 (38.7)	2 (22.2)	10 (45.5)

Variables are presented as either means (standard deviation (SD)) for continuous data or numbers (%) for categorical data. * Included symptomatic (stroke/TIA) and asymptomatic with significant lesions (>80% of diameter) in the carotid artery (noted in Doppler ultrasound examination); ** Defined if eGFR calculated by means of simplified (short) MDRD formula was below 60 mL/min/1.73 m^2^ BSA (body surface area). *Abbreviations*: ACS = acute coronary syndrome; CABG = coronary artery bypass grafting; CAD = coronary disease; CKD = chronic kidney disease; COPD = chronic obstructive; pulmonary disease; GI = gastrointestinal; PCI = percutaneous coronary intervention; PVD = peripheral vascular disease

**Table A2:** Results of laboratory studies and Doppler ultrasonography of Poznan Cardio severely sick male patients.

	All [*n* = 31]	Statin(−) [*n* = 9]	Statin(+) [*n* = 22]
WBC [10^9^/L]	8.02 (1.99)	8.70 (2.10)	7.74 (1.91)
Neutrophils	5.14 (1.50)	5.67 (1.88)	4.92 (1.29)
Lymphocytes	1.86 (0.66)	1.95 (0.79)	1.81 (0.62)
NLR	3.15 (1.69)	3.67 (2.79)	2.93 (0.99)
RBC [10^12^/L]	4.75 (0.30)	4.78 (0.3)	4.79 (0.31)
HGB [mM/L]	9.14 (0.74)	9.20 (1.05)	9.11 (0.60)
Platelets [10^9^/L]	250 (60)	251 (43)	250 (67)
PLR	154.0 (75.1)	157.5 (87.6)	152.5 (71.7)
Total cholesterol [mM/L]	4.49 (1.07)	4.66 (1.07)	4.42 (1.09)
LDL cholesterol	2.86 (1.21)	3.37 (1.12)	2.65 (1.20)
HDL cholesterol	1.12 (0.34)	1.11 (0.17)	1.12 (0.26)
Triglicerydes [mM/L]	1.90 (1.43)	1.64 (0.87)	2.0 (1.61)
Creatinine [mg/dL]	1.29 (0.80)	1.65 (1.38)	1.15 (0.32)
eGFR [mL/min/1.73 m^2^]	74.8 (26.7)	76.7 (39.6)	74.2 (20.5)
CRP [mg/L]	10.3 (3.4)	8.3 (4.2)	11.3 (2.9)
IMT [mm]	0.78 (0.16)	0.70 (0.12)	0.81 (0.16)

Variables are presented as means (SD). *Abbreviations*: CRP = C-reactive protein; eGFR = estimated glomerular filtration rate; HDL = high-density lipids; HGB = hemoglobin concentration; IMT = intima-media thickness; NLR = neutrophil-to-lymphocyte ratio; PLR = platelet-to-lymphocyte ratio; RBC = red blood cell count; WBC = white blood cells count.

## Appendix B. Assessment of Carotid Intima-Media Thickness (IMT)

The carotid IMT measures were performed with the use of high-quality sonography (System EPIQ 5, Philips N.V., The Netherlands) using the linear 12–3 MHz transducer. IMT was measured bilaterally at the distal wall of carotid arteries in length (cm) starting 1 cm below the carotid bulb. At least five thickness measurements were performed on each site, and its average value was accepted as the outcome.

## Appendix C. Data Management and Statistical Analysis

Continuous variables were validated for normality by means of the Shapiro–Wilk W test. If they satisfied criteria of normal distribution, they were expressed as means (SD).
